# High Chromosomal Reorganization and Presence of Microchromosomes in Chactidae Scorpions from the Brazilian Amazon

**DOI:** 10.3390/biology12040563

**Published:** 2023-04-07

**Authors:** Bruno Almeida, Stella Malcher, Marlyson Costa, Jonas Martins, Rudi Procópio, Renata Noronha, Cleusa Nagamachi, Julio Pieczarka

**Affiliations:** 1 Laboratório de Citogenética, Centro de Estudos Avançados da Biodiversidade, Instituto de Ciências Biológicas, Universidade Federal do Pará, Av. Perimetral da Ciência, km 01, Guamá, Belém 66075-750, PA, Brazil; 2 Instituto Federal de Educação, Ciência e Tecnologia do Pará-Campus Itaituba, R. Universitário, s/n, Maria Magdalena, Itaituba 68183-300, PA, Brazil; 3 Instituto Nacional de Pesquisas da Amazonia, Av. André Araújo, 2936, Petrópolis, Manaus 69067-375, AM, Brazil; 4 Medical School, Universidade do Estado do Amazonas, Av. Carvalho Leal, 1777, Cachoeirinha, Manaus 69065-170, AM, Brazil

**Keywords:** Scorpiones, monocentric chromosome, meiotic multi-chromosomal associations

## Abstract

**Simple Summary:**

Scorpions are excellent models for understanding the role of structural chromosomal rearrangements in genome evolution. However, for some families of Scorpiones, cytogenetic information is extremely limited. In the present study, we performed a karyotype study of four scorpions of the Chactidae family and found great chromosomal diversity in the genus *Brotheas*, including an intraspecific variation in *Brotheas amazonicus* (2n = 50 or 52). Changes in chromosome behavior during meiotic division and in the number and position of 45S rDNA sequence clusters were also recorded in *B. amazonicus*. In this article, we describe for the first time the occurrence of microchromosomes and bimodal karyotype in Scorpiones, and we propose hypotheses for their origin in *Neochactas parvulus*.

**Abstract:**

Scorpions are of particular interest in cytogenomic studies, as they can present a high incidence of chromosomal rearrangements heterozygous in natural populations. In this study, we cytogenetically analyzed four species of Chactidae. In *Brotheas*, 2n = 40 was observed in *Brotheas silvestris*, 2n = 48 in *Brotheas paraensis*, and 2n = 50 (cytotype A) or 2n = 52 (cytotype B) among populations of *Brotheas amazonicus*. Our results showed a bimodal karyotype in *Neochactas parvulus*, 2n = 54, with microchromosomes and a concentration of constitutive heterochromatin in macrochromosomes. The 45S rDNA is located in only one pair of the karyotype, with different heteromorphisms of clusters of this rDNA in the cytotype B of *B. amazonicus*, with NOR-bearing chromosomes involved in multi-chromosomal associations during meiosis I. The U2 snDNA was mapped in the interstitial region of distinct karyotype pairs of three Chactidae species. Our results indicate the possible formation of cryptic species in *B. amazonicus*; the different 45S rDNA configurations in the genome of this species may result from amplification and degeneration. We suggest that the bimodal karyotype in *N. parvulus* results from fusion/fission events and that the unequal distribution of repetitive DNAs between macro and microchromosomes contributes to the maintenance of its asymmetry.

## 1. Introduction

Scorpiones is one of the oldest orders of the phylum Arthropoda and comprises approximately 1900 species [1]. These arachnids are excellent models for the study of chromosomal rearrangements, as they have a high incidence of magnetic breaks and fusions, in addition to multiple reciprocal translocations that, during Meiosis I, are detected by the formation of multivalent associations [2,3,4]. These rearrangements in heterozygosity have great potential to promote gene expression modification, recombination expression and formation of reproductive barriers [5,6]. The factors that trigger this high karyotypic diversity in Scorpiones are still poorly known.

Different classes of repetitive DNAs may be involved in the genesis of chromosomal rearrangements, as they are enriched in unstable genomic regions, constituting sites susceptible to breakage [7,8]. Ectopic recombination involving repetitive sequences between non-homologous chromosomes, or transposition of mobile elements, can promote chromosomal translocations or inversions [9,10,11]. The 45S rDNA region comprises a set of tandem repeats composed of the 18S, 28S and 5.8S genes, separated by internal and external intergenic spacer regions, responsible for coding RNA components of the ribosomes [12,13]. Studies about the chromosomal mapping of this repetitive DNA in Scorpiones demonstrated that 45S rDNA is usually present in a karyotype pair [14,15,16,17]. However, in some species, extensive dispersal of this multigene has been recorded [18]. Interestingly, in many scorpions, the 45S rDNA carrier pair is involved in multi-chromosomal meiotic associations [3,18,19].

Fusion/fission events can originate bimodal karyotypes, which are characterized by the existence of classes of chromosomes with divergent sizes [20]. The origin of the bimodal karyotype can be related to fusion/fission-type chromosomal rearrangements, the hybridization process with subsequent polyploidy [21] or differential accumulation of repetitive sequences in certain chromosomes [22]. In bimodal karyotypes of some monocotyledonous plants and vertebrate animals, microchromosomes were recorded, which are extremely small in size, similar to dots, whose morphology is not possible to determine [21,23]. In these organisms, microchromosomes have a high gene content, replicate earlier in S phase than macrochromosomes, and are typically observed in the central region of the interphase nucleus [24,25,26,27].

In Scorpiones, two basic chromosome types are observed: holocentric (with kinetochore proteins along its length) in Buthidae and monocentric (with centromere restricted to a certain region of the chromosome) in other families [28]. Chromosomal rearrangements in the heterozygous state have been reported in both cases, despite being more frequently recorded in Buthidae [17,29]. Cytogenetic data from scorpions carrying monocentric chromosomes are limited, and in some families, only a few species have been investigated. In Chactidae, for example, whose geographic distribution extends along the American continent and encompasses 15 genera and 208 species, only *Brotheas amazonicus* had a limited description of its karyotype [30]. In the present study, we cytogenetically analyzed four species of Chactidae, belonging to the genera *Brotheas* and *Neochactas*, in order to infer the chromosomal rearrangements responsible for the evolution of the karyotype in this family and the role of 45S rDNA in this process. Additionally, we characterize a bimodal karyotype in *Neochactas parvulus*, the first described for the order Scorpiones.

## 2. Materials and Methods

### 2.1. Sampling and Karyotype Analysis

Data on the number and sex of individuals sampled in the present study, as well as the respective collection locations, are described in Table 1. Taxonomic identification was performed according to Lourenço [31]. The specimens were deposited in the collection of the Laboratory of Medical Entomology and Venomous Arthropods (LEMAP/UFPA). Gonads and embryos were hypotonized in 0.075M KCl and subsequently fixed in a methanol:acetic acid (3:1) solution. Cell suspension generated from the digestion of gonads and embryos in 60% acetic acid was spread on slides at 45 °C. Chromosomes were stained with 5% Giemsa. C banding was performed according to Sumner [32]. Chromosomal measurements were performed using the DRAWID software [33]. The determination of chromosomal morphology followed the classification by Levan et al. [34] and was based on I metaphases, in which the centromere is more easily visualized after C-banding.

### 2.2. Probes

Probe 45S rDNA was produced from plasmid pTa71, which contains the complete sequence of this *Triticum aestivum* rDNA [35]. In turn, arthropod telomere sequences and U2 snDNA were obtained by Polymerase Chain Reaction (PCR) using previously described primers [36,37], respectively, and genomic DNA from *B. amazonicus*. The PCR reactions consisted of: 16.25 µL of sterile water, 2.5 µL of 10x Taq Polymerase buffer, 2 µL of DNTP (2 mM), 1 µL of genomic DNA (100 ng), 1 µL of MgCl2 (50 mM), 1 µL of each forward and reverse primer (10 mM), and 0.25 µL of 1U Taq Polymerase. The thermal configurations of the PCRs were: 1 cycle of 94 °C (5 min); 35 cycles of 94 °C (1 min), 53 to 55 °C (1 min, normally 53 °C for U2 snDNA and 55 °C for telomeric repeats) and 72 °C (1 min); 1 cycle of 72 °C (10 min); and 1 cycle 4 °C (hold). The U2 snDNA PCR product was cloned into the pGEM-T vector (Promega^®^) and transformed into the bacterium *Escherichia coli* DH5α (Gibco GBRL^®^). All probes were produced by nick and translated using digoxigenin-14-dUTP (Roche, Mannheim, Germany).

### 2.3. Fluorescent In Situ Hybridization (FISH)

FISH was performed according to previously established protocols for arthropods [38]. Initially, the chromosomes were treated with a 1% Pepsin solution, fixed in 4% Paraformaldehyde, and dehydrated in an alcohol battery (70%, 90% and 100%). Denaturation of chromosomal DNA and probes occurred at 70 °C and 100 °C, respectively. Slides were kept overnight at 37 °C for hybridization. Subsequently, slides were washed with 2xSSC and 4xSSC-Tween to remove nonspecific hybridizations. Probes were detected with Anti-digoxigenin-FITC. Chromosomes were counterstained with 4-6-diamidino-2-phenylindole (DAPI) containing antifading VECTASHIELD (Vector).

## 3. Results

### 3.1. Karyotype and Repetitive DNA Mapping in Brotheas

**Figure 1:**
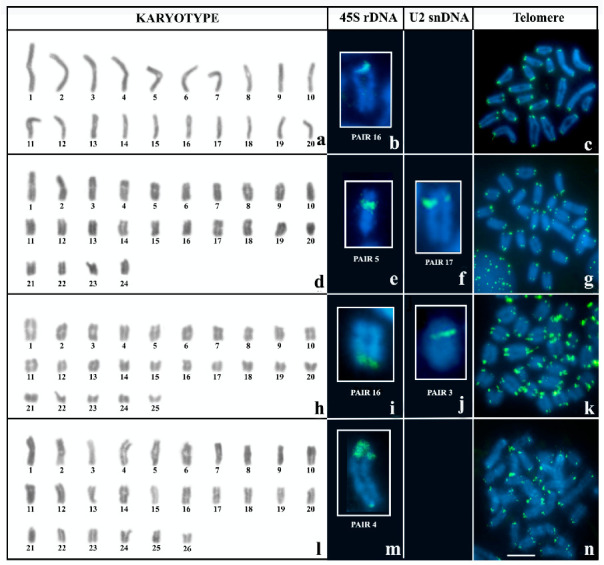
Meiotic karyotypes (**a**,**d**,**h**,**l**) and FISH with probes 45S rDNA (**b**,**e**,**i**,**m**), U2 snDNA (**f**,**j**) and TTAGG telomeric repeats (**c**,**g**,**k**,**n**) in different species of *Brotheas*. (**a**–**c**) *B. silvestris*, 2n = 40; (**d**–**g**) *B. paraensis*, 2n = 48; (**h**–**k**) *B. amazonicus* cytotype A, 2n = 50; (**l**–**n**) *B. amazonicus* cytotype B, 2n = 52. Barr = 10 μm.

**Figure 2:**
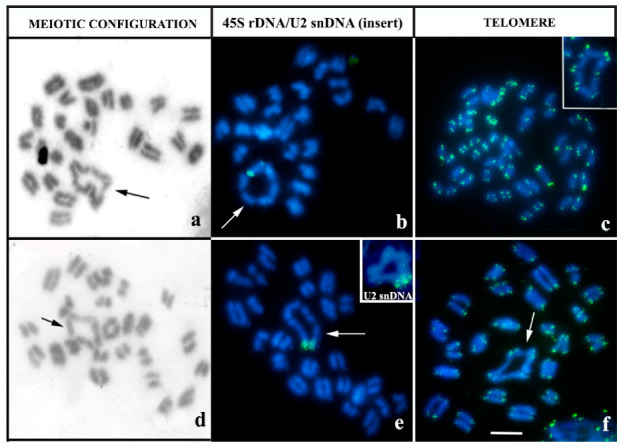
Multi-chromosomal meiotic associations in individuals of *B. amazonicus* cytotype B: (**a**) Hexavalent (arrow) stained-giemsa; (**b**) FISH with 45S rDNA probe in hexavalent (arrow); (**c**) FISH with telomeric probe in individual with hexavalent (insert); (**d**) quadrivalent (arrow) stained-giemsa; (**e**) FISH with 45S rDNA probe (arrow) and U2 snDNA (insert) in quadrivalent; (**f**) FISH with telomeric probe in individual with quadrivalent. Bar = 10 μm.

Typical monocentric chromosomes were recorded in all analyzed species. In the sample of males, chromosomes with differential meiotic behavior that would suggest the occurrence of heteromorphic allosomes were not observed (Figure 1). In *Brotheas*, the diploid number and the karyotype formula varied according to the species: *Brotheas silvestris* had 2n = 40 and a karyotype consisting of 28 metacentric, 6 submetacentric and 6 acrocentric (Figure 1a); *Brotheas paraensis* showed 2n = 48 and karyotype with 14 metacentric, 4 submetacentric and 30 acrocentric (Figure 1d); *Brotheas amazonicus* presented two distinct karyotypes, 2n = 50 (24 metacentric, 4 submetacentric and 22 acrocentric) in the population from Manaus-cytotype A (Figure 1h) and 2n = 52 (20 metacentric, 10 submetacentric and 22 acrocentric) in the sample from Parintins-cytotype B (Figure 1l).

Different 45S rDNA distributions were registered among the analyzed karyotypes. In *B. silvestris,* this sequence was observed in the terminal region of the short arm of pair 16 (Figure 1b). In *B. paraensis*, 45S rDNA is located in the proximal region of the short arm of pair 5 (Figure 1e). In *B. amazonicus,* two distinct patterns were evidenced: in cytotype A (2n = 50), 45S rDNA was mapped in the terminal region of the long arm of pair 16 (Figure 1i); on the other hand, in cytotype B of *B. amazonicus* (2n = 52), carriers of 26 bivalents, this repetitive DNA was observed in pair 4, with one of the chromosomes of this pair carrying only one signal at one end, while its homolog had two 45S rDNA clusters of unequal sizes at both ends (Figure 1m).

Mapping of the U2 snDNA gene was successfully obtained in only two species of *Brotheas*. In *B. paraensis* U2 snDNA is located in the interstitial region of the short arm of pair 17 (Figure 1f). In *B. amazonicus* cytotype A (2n = 50), this multigene was observed in the short arm of pair 3 (Figure 1j).

Arthropod telomere sequences (TTAGG) were recorded at the ends of chromosomes in all individuals studied (Figure 1c,g,k,n).

### 3.2. Multi-Chromosomal Meiotic Associations in Brotheas

Metaphase I cells from male individuals of the four species did not show any evidence of chiasm (achiasmatic meiosis) (Figure 1). In most specimens, the following amounts of homologous pairs with regular meiotic behavior were observed: 20 bivalents in *B. silvestris* (Figure 1a), 24 bivalents in *B. paraensis* (Figure 1d) and 25 bivalents in *B. amazonicus* cytotype A (2n = 50) (Figure 1h). In contrast, in *B. amazonicus* cytotype B (2n = 52), different meiotic configurations were observed: 26 regular bivalents in 5 individuals (Figure 1l); 23 bivalents and 1 hexavalent in 2 individuals (Figure 2a); and 24 bivalents and 1 quadrivalent in 1 individual (Figure 2d).

FISH in meiotic cells of this cytotype also revealed that specimens with multivalents present a chromosomal chain with the pair carrying the 45S rDNA: in specimens carrying the hexavalent, two 45S rDNA clusters of unequal sizes were observed in a single component chromosome of this multivalent, one at each end (Figure 2b). The individual that had a quadrivalent showed that this rDNA is present only in a terminal region of two heteromorphic elements involved in this association (Figure 2e). In the *B. amazonicus* cytotype B individual (2n = 52) carrying the quadrivalent, U2 snDNA was recorded in the terminal region of a pair involved in this association (Figure 2e, see insert).

Arthropod telomere sequences (TTAGG) were recorded at the ends of chromosomes in all individuals studied (Figure 2c,f).

### 3.3. Neochactas parvulus

**Figure 3:**
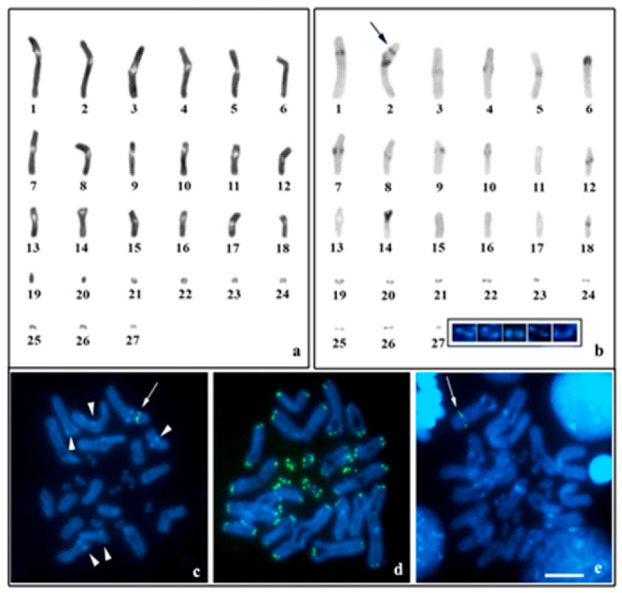
Karyotype of *N. parvulus*: (**a**) conventional staining with Giemsa, note the difference in size between microchromosomes and macrochromosomes; (**b**) C banding, the arrow on pair 2 indicates the additional C-band on the short arm. In the insert, some specimens of microchromosomes subjected to DAPI staining after C-banding; (**c**) 45S rDNA; DAPI+ heterochromatic blocks are indicated by arrowheads; (**d**) TTAGG repeats. (**e**) U2 snRNA. Bar = 10 μm.

*N. parvulus* presented monocentric chromosomes and diploid number 2n = 54 (Figure 3); the karyotype of this species is highly bimodal and consists of 4 metacentric, 26 submetacentric, 6 acrocentric and 18 microchromosomes, with no heteromorphic sex pairs (Figure 3a).

In *N. parvulus*, c-banding with subsequent DAPI staining showed strong bands of heterochromatin rich in AT base pairs in the pericentromeric region of most macro and microchromosomes, except in pairs 11, 13, 15–17, 23, 25 and 27 (Figure 3b). An additional C-band was recorded in the interstitial region of the short arm of pair 2 (Figure 3b). The 45S rDNA is found in the interstitial region of the short arm of pair 2 and is co-located with the C-band observed in the same region (Figure 3c). Arthropod telomere sequences (TTAGG) were recorded at the ends of chromosomes in all individuals studied (Figure 3d). The U2 snDNA gene was recorded in the interstitial region of the long arm of pair 6 (Figure 3e).

**Figure 4:**
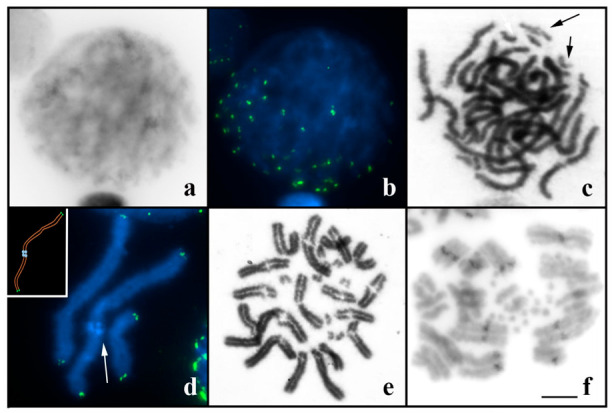
Meiotic behavior in *N. parvulus*: (**a**) Zygotene. (**b**) The same nucleus in “a” subjected to FISH with a telomere probe; note the polarized arrangement of the telomeres. (**c**) Pachytene; arrows indicate microbivalents. (**d**) Isolated pachytene bivalents; the arrow indicates heterochromatic domains in the pericentromeric region. The insert shows a schematic representation of the bivalent indicated by the arrow. (**e**) Post-pachytene. (**f**) Anaphase I. Bar = 10 μm.

Meiotic analysis revealed regular synaptic behavior for all chromosome pairs of *N. parvulus*. During zygotene, chromosomes assemble into a configuration with telomeres clustered at a pole of the cell nucleus (Figure 4a,b). In pachytene, all bivalents (macro and microchromosomes) are visualized in the form of double filaments with complete synapses (Figure 4c). The degree of extension of chromosomal fibers during pachytene allowed us to observe the presence of two heterochromatic domains in the pericentromeric region of some bivalents subjected to c-banding (Figure 4d). Post-pachytene cells showed 27 highly condensed and paired bivalents without any evidence of chiasmata (Figure 4e). From this phase until Metaphase I, the pericentromeric region was asynaptic (Figure 4e). In the metaphase I/anaphase I transition, the components of the bivalents begin disjunction (Figure 4f).

## 4. Discussion

### 4.1. Comparative Karyotype Analysis of B. silvestris and B. paraensis

Our results demonstrated high cytogenetic diversity among members of the genus *Brotheas* from the Brazilian Amazon. Unfortunately, there is no phylogenetic information for *Brotheas* available in the current literature, which makes it impossible to generate hypotheses of chromosomal evolution for this group. Among the studied species, *B. paraensis* and *B. silvestris* are sympatric and morphologically related. Lourenço [31], in his taxonomic key, differs these two species only in relation to coloration (brown to blackish-brown in *B. paraensis* and reddish to reddish-brown in *B. silvestris*), size (40–50 cm in *B. paraensis* and 55–70 cm in *B. silvestris*) and level of granulation in some body segments. However, these characters can be highly polymorphic, as previously observed in other species [31], making it difficult to differentiate these scorpions. Our findings allowed distinguish them at the cytogenetic level (2n = 48 in *B. paraensis* and 2n = 40 in *B. silvestris*), proving to be an excellent tool for the taxonomic identification of both. Comparison of the karyotypic formula between the *B. paraensis* e *B. silvestris* also allowed inferring the occurrence of inversions between both; this rearrangement has been recurrently documented in other scorpions with monocentric chromosomes, such as *Urodacus* [39], *Euscorpius* [17] and *Hadogenes* [15]. Inversions may also explain the divergence in 45S rDNA physical location between *B. paraensis* (proximal NOR) and *B. silvestris* (terminal NOR).

### 4.2. Cytogenetic Diversity in B. amazonicus

**Figure 5:**
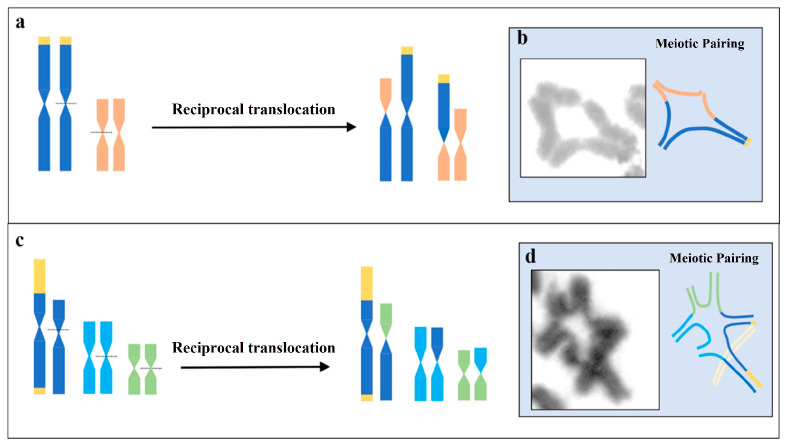
Schematic interpretation for observed translocations in *B. amazonicus* cytotype B: (**a**) origin and (**b**) metaphase I pairing of the quadrivalent association; (**c**) origin and (**d**) metaphase I pairing of the hexavalent association. Yellow regions in the schemes correspond to 45S rDNA sites.

In *B. amazonicus*, the 2n = 50 recorded for cytotype A agrees with the data presented by Ferreira [30]. This cytotype showed profound differences in karyotypic structure in relation to cytotype B (2n = 52), suggesting the possible existence of cryptic species. In the specific case of cytotype B, the occurrence of meiotic multivalents showed that multiple translocations are important mechanisms occurring in chromosomal differentiation in relation to cytotype A (see Figure 5). Meiotic chains have been recorded in several families of Scorpiones and, in general, promote heteromorphisms in several karyotype pairs [2,4,28,29]. Ecological data on *B. amazonicus* revealed that this species has low motility (except for males during the reproductive period), high abundance in certain regions of the Amazon Forest, forming small groups in places with a large number of hiding places, such as termite mounds and fallen logs [40]. These population attributes may promote a high incidence of mating between individuals heterozygous for different translocations [4,29] and contribute to an increase in the rate of occurrence of multivalents in the B cytotype of *B. amazonicus*.

In cytotype B of *B. amazonicus*, analysis by FISH showed the presence of two clusters of 45S rDNA heteromorphic in size, located in pair 4 (Figure 1m). Furthermore, in the larger homolog of this pair, an extra site at the opposite end was recorded. This result can be explained by the amplification of 45S rDNA motifs, with subsequent uneven crossing-over between them, similar to that observed in fish [41], amphibians [42], plants [43] and other arthropods [15,44]. The amplification of 45S rDNA is a phenomenon epigenetically controlled by the cell and may constitute a response to environmental factors [45]. In some organisms, such as plants [46], mammals [47] and fish [41], it has been demonstrated that 45S rDNA constrictions constitute sites prone to breakage and can promote genomic instability. During the meiotic bouquet, the chromosome ends and joins the nuclear membrane, and the telomeres remain clustered at a single pole of the cell. According to Hirai [48], several associations between terminal 45S rDNA clusters and heterochromatic regions may occur in this phase of the meiotic cycle. Thus, it is plausible to suggest that the extra site observed in this cytotype probably originated through the translocation of part of the repeats of the main clusters during the organization of the bouquet in prophase I.

In cytotype B of *B. amazonicus*, although 45S rDNA clusters have differences in size, our results demonstrated regular pairing between them during meiosis I (Figure 1m). In individuals who have the hexavalent, the pairing occurred between a 45S rDNA cluster and euchromatic regions (which do not carry this repetitive DNA), characterizing a case of heterosynapse (Figure 2b and Figure 5). This meiotic mechanism has been widely documented in species heterozygous for multiple reciprocal translocations, such as amphibians [49], mammals [50] and insects [51]. In this case, the synaptonemal complex can be formed in repetitive regions without the need for homology [52], with adjustment in the size of the chromosomes by equalizing the synapsed axial elements [53].

**Figure 6:**
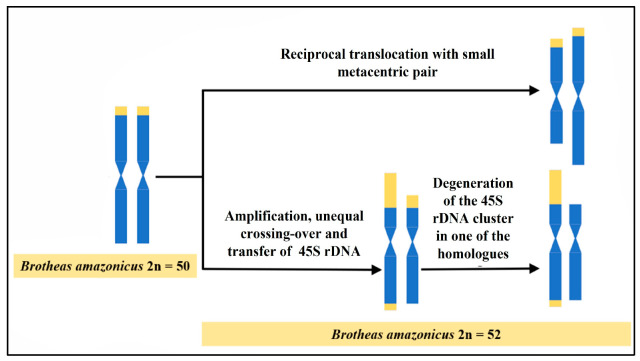
Hypothesis for the evolution of 45S rDNA clusters in *B. amazonicus* cytotypes.

Considering the 45S rDNA distribution observed in the present study, we propose that the multi-chromosomal associations recorded in the B cytotype of *B. amazonicus* are of independent origin (see Figure 6). In the specific case of the specimen carrying the quadrivalent (cytotype B), the origin of this meiotic configuration may be related to the occurrence of a reciprocal translocation between the NOR pair and an unidentified small metacentric pair; this hypothesis is supported by the existence of terminal 45S rDNA clusters and similar among homologs, located only at one end of one of the pairs involved in this association. On the other hand, we hypothesize that in the specimens that have hexavalent, the pair that presents the NOR may have been derived from the pair 4 (carrier of the rDNA) typical of most individuals of the B cytotype of *B. amazonicus*, previously described. As part of the concerted evolution of this repetitive DNA, some 45S rDNA repeats may have degenerated via recombination in order to promote the homogenization of such sequences [54,55]. Thus, in individuals who presented the hexavalent, this mechanism can be evoked to explain the presence of only one chromosome carrying the 45S rDNA. Later, reciprocal translocations involving this homolog with other karyotype pairs resulted in the formation of the hexavalent. Interstitial telomere sequences (ITS) were not observed in any meiotic multivalent, suggesting that translocations would not involve telomeres of the original chromosomes or that ITSs may have been removed after the occurrence of these rearrangements [56].

### 4.3. Bimodal Karyotype in N. parvulus

Bimodal karyotypes are rare in invertebrates, sporadically recorded in members of the phylum Arthropoda [57,58]. In some orders of Insecta, bimodality is characterized only by the presence of a pair of giant sex chromosomes [59] or by the formation of a single pair of microchromosomes [60]. Similar results were found by Shanahan [39], who observed extremely small telocentric chromosomes in the scorpion *Urodacus manicatus* (Urodacidae). The absence of cytogenetic data from other species of the genus *Neochactas* makes it impossible to reconstruct the karyotypic evolution of *N. parvulus*. Nevertheless, the mapping of the 45S rDNA and U2 snDNA multigene families in *N. parvulus* allows us to discard the hypothesis of the origin of this karyotype by allopolyploidy since only one site of each of these sequences was observed in the complement of this species. On the other hand, the unimodal karyotypes of *Brotheas* and other scorpions allow us to suggest that fusion/fission events were the main responsible for the origin of bimodality in *N. parvulus*. In this case, chromosomes of regular size would result from fusions, while microchromosomes would originate through fissions [21]; this phenomenon is widely documented in other animal groups that present bimodal karyotypes, such as Testudines [61], Aves [62] and Squamata [63]. High fusion/fission rates have been revealed in scorpions with holo and monocentric chromosomes [3]. In the latter, these alterations have been documented mainly in the genera *Urodacus* [39], *Euscorpius* [17], *Hadogenes* [15] and *Heterometrus* [2]. According to Imai [64], fissions can be positively selected to resolve interlocks and ectopic crossing-overs formed inside the cell nucleus, preventing the formation of translocations and inversions. Considering this information, it is possible that *N. parvulus* microchromosomes originated from the fissions of large chromosomes in order to avoid the deleterious effects of rearrangements in heterozygosity.

In most animals, microchromosomes are kept synapsed during pachytene and carry out crossing-over in this phase of meiosis I [60,65] since the formation of chiasmata is necessary for their stability during meiotic division. On the other hand, in several achiasmatic lineages of Heteroptera, microchromosomes synapse late in pachytene, are univalent in diplotene (due to the absence of chiasmata), and later form a pseudo-bivalent in diakinesis [66]. Similarly, males of *N. parvulus* analyzed in the present study are achiasmatic. However, in *N. parvulus*, microchromosomes remained in the bivalent form until the onset of anaphase I (see Figure 4f). This result can be explained by the presence of a modified synaptonemal complex that remains until the late stages of prophase I, as recorded in other scorpions [16,28,67]. Thus, in *N. parvulus,* this meiotic adaptation may replace the function of chiasmata and allow normal disjunction of microbivalents during anaphase I.

Several studies have shown that the recombination rate tends to differ between the chromosome sets of a bimodal karyotype over time [22,68]. This characteristic is necessary to maintain the asymmetry of a bimodal karyotype after its formation [69]. In *N. parvulus*, this hypothesis cannot be fully applied, as meiosis is achiasmatic in this species, suggesting a low crossing-over rate or its total absence in all pairs of chromosomes in prophase I. Thus, the persistence of asymmetry in the bimodal karyotype of this scorpion must be related to other mechanisms. For some groups of plants, it is suggested that the amplification of chromosome-specific heterochromatic repetitive DNAs contributes to an increase in the difference in size between the classes of the bimodal karyotype [22,69]. Interestingly, in *N. parvulus*, we observed that chromosomal pairs of regular size have a higher amount of constitutive heterochromatin (CH) in relation to microchromosomes. This pattern is unusual for monocentric Scorpiones chromosomes, which have low heterochromatic content, generally restricted to small pericentromeric blocks [2,28,39,70,71]. Thus, we suggest that CH amplification in some karyotype pairs of *N. parvulus* may contribute to the maintenance of the bimodality of the karyotype of this species.

## 5. Conclusions

In this study, we describe for the first time cytogenetic data for members of the Chactidae family. Two distinct karyotypes were observed in *B. amazonicus*, originating mainly through inversions and reciprocal translocations, suggesting the occurrence of cryptic speciation. Additionally, extensive variation in the position and size of 45S rDNA clusters and the presence of multi-chromosomal meiotic associations were recorded in the B cytotype of *B. amazonicus*. The bimodality of the *N. parvulus* karyotype with 18 microchromosomes that remain synaptic during meiosis I was also recorded. We conclude that microchromosomes in *N. parvulus* originate from fission events and that the accumulation of heterochromatic repetitive DNAs in macrochromosomes may contribute to the maintenance of the bimodal karyotype in this scorpion.

## Figures and Tables

**Table 1:** General data about the sample used in this study.

Genus	Species	*N*	Locality	Geographic Coordinate
*Brotheas*	*B. amazonicus*	1 male, 1 female, 16 embryos	Manaus/AM	3°06′10″ S/59°58′42″ O
		7 males, 5 females, 9 embryos	Parintins/AM	2°38′15″ S/56°43′46″ O
	*B. silvestris*	3 males, 1 female, 19 embryos	Óbidos/PA	1°54′16″ S/55°31′12″ O
	*B. paraensis*	2 males, 1 female, 12 embryos	Óbidos/PA	1°54′16″ S/55°31′12″ O
*Neochactas*	*N. parvulus*	2 males, 1 female	Óbidos/PA	1°54′16″ S/55°31′12″ O

## Data Availability

All data presented here are included in the manuscript. Additional questions should be directed to the corresponding author.
